# ER and vacuoles: never been closer

**DOI:** 10.3389/fpls.2014.00020

**Published:** 2014-02-04

**Authors:** Corrado Viotti

**Affiliations:** Umeå Plant Science Centre, Department of Plant Physiology, Umeå UniversityUmeå, Sweden

**Keywords:** endoplasmic reticulum, COPII vesicles, Golgi apparatus, trans-Golgi network, multivesicular body, vacuole

## Abstract

The endoplasmic reticulum (ER) represents the gateway for intracellular trafficking of membrane proteins, soluble cargoes and lipids. In all eukaryotes, the best described mechanism of exiting the ER is via COPII-coated vesicles, which transport both membrane proteins and soluble cargoes to the *cis*-Golgi. The vacuole, together with the plasma membrane, is the most distal point of the secretory pathway, and many vacuolar proteins are transported from the ER through intermediate compartments. However, past results and recent findings demonstrate the presence of alternative transport routes from the ER towards the tonoplast, which are independent of Golgi- and post-Golgi trafficking. Moreover, the transport mechanism of the vacuolar proton pumps VHA-a3 and AVP1 challenges the current model of vacuole biogenesis, pointing to the endoplasmic reticulum for being the main membrane source for the biogenesis of the plant lytic compartment. This review gives an overview of the current knowledge on the transport routes towards the vacuole and discusses the possible mechanism of vacuole biogenesis in plants.

## ENDOPLASMIC RETICULUM: ENTRANCE TO THE SECRETORY PATHWAY

The endoplasmic reticulum (ER) consists on a network of interconnected membrane tubules and cisternae (“reticulum”) stretching across the entire cytoplasm (“endoplasmic”). First discovered in culture cells from chicken embryos (Porter et al., 1945), the ER is present in all eukaryotic cells, and is the intracellular compartment where membrane proteins, soluble cargoes and lipids are synthesized. From the ER, correctly folded membrane and soluble proteins are transported to other endomembrane compartments or to the extracellular space along the secretory pathway (Vitale and Denecke, 1999). For all eukaryotes, the best characterized mechanism of exiting the ER is the COPII-mediated transport. The coat protein complex II (COPII) assembles on specific locations of the ER membrane, called ER-exit sites (ERES), from which COPII-coated vesicles bud off. The assembly of COPII begins with the activation of the small guanosine triphosphatase (GTPase) SAR1 provided by the ER membrane-bound guanine nucleotide exchange factor (GEF) SEC12, which leads to the coordinated recruitment of the cytosolic heterodimers SEC23/SEC24 and SEC13/SEC31 to the ERES (Nakano et al., 1988; Barlowe and Schekman, 1993; Barlowe et al., 1994). Cargo recognition is provided by SEC24 and SAR1, whereas multiple adjacent SEC13/SEC31 subcomplexes drive the bending of the ER membrane using the energy of GTP hydrolysis (Brandizzi and Barlowe, 2013). Passive incorporation of soluble cargoes into COPII vesicles can occur (Wieland et al., 1987; Denecke et al., 1990; Matsuoka and Nakamura, 1991; Phillipson et al., 2001; Thor et al., 2009), instead membrane proteins and receptors require di-acidic or di-hydrophobic motifs in their cytosolic domains for efficient transport (Kappeler et al., 1997; Nishimura and Balch, 1997; Contreras et al., 2004; Hanton et al., 2005). In mammals, most COPII subunits have one or more paralogs, which generate a robust repertoire of COPII-coated vesicles with tissue specificities and selectivity for different cargo molecules (reviewed in Zanetti et al., 2011). In plants much less is known about specificities among different COPII-coated carriers, even though it has been recently shown that the concomitant function of all three SEC24 members of *Arabidopsis* is necessary for the development of the gametophytes (Conger et al., 2011; Tanaka et al., 2013). After a long debate whether COPII vesicles versus COPII-coated tubules existed in plant cells, ultrastructural analysis of high-pressure frozen samples and 3D tomography reconstructions have shown that COPII vesicles are present also in plants (Ritzenthaler et al., 2002; Donohoe et al., 2007; Robinson et al., 2007; Kang and Staehelin, 2008).

## LYTIC VACUOLES

The plant lytic vacuole can occupy up to 90% of the total volume in mature vegetative cells. Its remarkable size allowed Antonie van Leeuwenhoek to notice the vacuole already in the 1670s, at the dawn of microscopy. The name “vacuole” was coined from “vacuum,” because Felix Dujardin, in 1872, thought he was facing an empty space (Leigh and Sanders, 1997; De, 2000). On the contrary, the vacuolar content can generate a stationary turgor pressure of up to five bars (Zimmermann et al., 1980), which provides the driving force for plants’ growth by pushing the cells to expand in oriented directions. Moreover, the lytic vacuole plays a crucial role in pH homeostasis, storage of ions, degradation of cellular waste, defense against pathogens, and in buffering abiotic stresses. The rapid release from or uptake to the vacuolar lumen of ions and water allow plants to efficiently cope with diversified environmental challenges. The multiple roles of plant lytic vacuoles are regulated by the activity of transporters that use the energy of the electrochemical gradient generated across the tonoplast by the vacuolar H^+^-ATPase (V-ATPase) and vacuolar H^+^-PPase (V-PPase). Despite good knowledge of the biochemistry and function of the vacuolar proton pumps (Maeshima, 2001; Schumacher and Krebs, 2010), little is known about the mechanisms of their sorting and the intracellular routes they follow to reach the tonoplast. However, recent data has shown that both the V-ATPase and V-PPase of *Arabidopsis* are incorporated to the tonoplast via a novel mechanism that also challenges the current model for vacuole biogenesis (Viotti et al., 2013).

## GOLGI- AND POST-GOLGI-MEDIATED TRANSPORTS TO THE LYTIC VACUOLE

**FIGURE 1:**
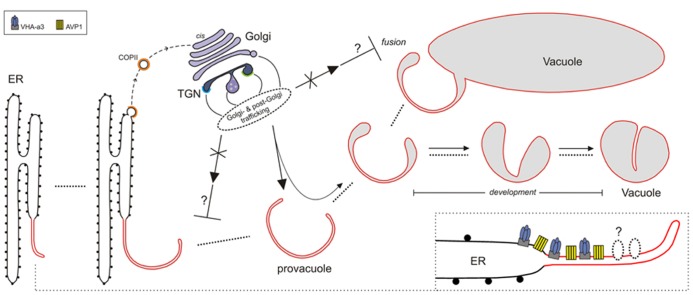
**Model for lytic vacuole biogenesis in *Arabidopsis*.** The precursors of vacuoles, the provacuoles, arise from the endoplasmic reticulum (ER) via maturation. The vacuolar proton pumps VHA-a3 and AVP1 (and perhaps other tonoplast proteins) are incorporated to the nascent provacuole directly from the ER-membrane through an uncharacterized mechanism (lower right corner). Golgi- and post-Golgi trafficking (solid arrows) continuously contribute, during the different steps of vacuole formation (dotted lines), to the development of the vacuolar lumen via the delivery of soluble cargoes (e.g., acid proteases) and tonoplast-resident proteins that exit the ER with COPII-coated vesicles (dashed arrow). Provacuoles can also fuse with already developed vacuoles (*fusion*). Impairment of Golgi- and post-Golgi trafficking (crossed arrows) leads to aberrant multilayered provacuoles.This observation could be explained if impairment of Golgi- and post-Golgi trafficking would interfere (⊥) with the process of provacuoles-release from the ER, or with the fusion between provacuoles and vacuoles.

Tonoplast-resident proteins and vacuolar soluble cargoes are synthesized in the ER, many of them are delivered to the *cis*-side of the Golgi apparatus via COPII vesicles, and from the Golgi they proceed further through the secretory pathway (**Figure 1**; Pedrazzini et al., 2013; Xiang et al., 2013). Vesicle transport between endomembrane compartments is mediated by different effector molecules, among which are the Rab GTPases, that are members of the ras superfamily of regulatory GTPases (Rutherford and Moore, 2002). The dissection of distinct steps of vacuolar transport using nucleotide-deficient mutants of different Rab GTPases in tobacco leaf epidermis cells has shown that tonoplast-resident proteins might follow at least three different routes (Bottanelli et al., 2011). In agreement with this finding, it has been shown that the sucrose transporter SUC4 and the *myo*-inositol transporter INT1 of *Arabidopsis* are delivered to the tonoplast in an adaptor protein complex 3 (AP3)-dependent and -independent manner respectively (Wolfenstetter et al., 2012). AP complexes sort cargo proteins into coated vesicles, and AP3 is involved in vacuolar trafficking. The exact localization of AP3 in plants is uncertain, because this adaptor seems to interact with clathrin (Lee et al., 2007; Zwiewka et al., 2011), which is present at the *trans*-Golgi network (TGN; Kang et al., 2011), whereas SUC4 accumulates at the Golgi apparatus in protoplasts isolated from *ap3* mutant seedlings, suggesting a Golgi-derived vesicle transport (Wolfenstetter et al., 2012).

From the TGN other two clathrin-mediated vacuolar transport carriers have been proposed to exist. Each of them has an EPSIN N-TERMINAL HOMOLOGY (ENTH) protein, EPSIN1 or MTV1 respectively, that acts as a monomeric adaptor for clathrin recruitment. Both EPSIN1 and MTV1 localize to the TGN, and both the respective knock-out mutants show defects in vacuolar transport (Song et al., 2006; Sauer et al., 2013).

The last and best known carrier in the vacuolar branch of the secretory pathway is the multivesicular body (MVB), which is an independent organelle (Tse et al., 2004) that arises from the TGN through a maturation process that involves the function of the TGN-located vacuolar proton pump VHA-a1, the calcium dependent phospholipid binding protein ANNEXIN 3, and the ESCRT-machinery (Scheuring et al., 2011). As in yeast and mammals, plant membrane proteins destined for degradation due to physiological turnover are incorporated to the MVB’s intraluminal vesicles through the function of the ESCRT complexes, and then released inside the vacuole via MVB-to-vacuole fusion (Reichardt et al., 2007; Spitzer et al., 2009; Viotti et al., 2010; Scheuring et al., 2011). Soluble vacuolar cargoes dissociate from vacuolar sorting receptors (VSRs) in an acidic environment (Kirsch et al., 1996), therefore this event might occur in the lumen of the TGN, which is the most acidic organelle among the intermediate compartments (Martinière et al., 2013; Shen et al., 2013). Soluble cargoes are then incorporated into the lumen of nascent MVBs, that arise from the TGN (Scheuring et al., 2011), for vacuolar transport. However, VSRs are localized both to the TGN and MVBs (Niemes et al., 2010; Stierhof and El Kasmi, 2010; Viotti et al., 2010), and the location from where they recycle is a matter of controversy (De Marcos Lousa et al., 2012; Robinson et al., 2012). An example of soluble cargo transported via MVBs is the cysteine protease aleurain (Miao et al., 2008), while regarding tonoplast resident proteins it has been recently shown that the auxin transporter WAT1 additionally co-localizes with the late endosomal marker RabG3f (Ranocha et al., 2013).

## UNCONVENTIONAL ER-EXPORT OF PROTEINS TO THE VACUOLE

The conventional transport of proteins to the vacuole involves COPII-mediated ER-exit and the passage through several intermediate steps and compartments. Hence, the vacuole, together with the plasma membrane, may be seen as the most distal point of the secretory pathway.

However, it was shown by Gomez and Chrispeels (1993) that the tonoplast intrinsic protein α-TIP, unlike the soluble vacuolar protein phytohemagglutinin (PHA), can reach the lytic vacuole even after brefeldin A (BFA) or monensin treatment when transiently expressed in tobacco leaves, suggesting the presence of different vacuolar transport routes. Few years later, Jiang and Rogers (1998) showed that a chimera composed by the C-terminal domain of α-TIP fused to the transmembrane domain of the VSR BP80 reaches the protein storage vacuole (PSV) through a direct route from the ER. Evidence for an alternative mechanism of vacuolar trafficking was provided by the analysis of the calcineurin B-like (CBL) proteins, which are calcium sensors functioning in different locations within a cell (Batistivč and Kudla, 2009). Among the ten members of *Arabidopsis*, CBL2, CBL3, CBL6 and CBL10 are targeted to the tonoplast in a COPII-independent manner, since overexpression of a dominant-negative mutant of SAR1did not interfere with their localization (Batistivč et al., 2010). Moreover, it was shown that CBL6 is transported to the vacuole bypassing both the Golgi and post-Golgi compartments (Bottanelli et al., 2011). CBL proteins, however, seem not to enter the secretory pathway, but are rather synthesized in the cytosol and delivered to the tonoplast due to the presence of a tonoplast targeting signal (TTS) in their N-terminal domain (Bottanelli et al., 2011; Batistivč et al., 2012; Tang et al., 2012). An example of soluble cargo transported to the plant vacuole through an unconventional route is the human α-mannosidase MAN2B1, which still reaches the vacuolar lumen even upon BFA treatment when transiently expressed in tobacco leaf mesophyll protoplasts (De Marchis et al., 2013).

The most abundant tonoplast resident protein, the vacuolar H^+^-ATPase VHA-a3, is transported to the vacuole through a novel mechanism. By blocking COPII-mediated transport via BFA treatment of GNL1 BFA-sensitive *Arabidopsis* seedlings (Richter et al., 2007), VHA-a3 was not retained in the ER and was detected as normal at the tonoplast, whereas the TGN-located H^+^-ATPase VHA-a1 was efficiently retained in the endoplasmic reticulum, indicating that VHA-a3 exits the ER in a COPII-independent manner (Viotti et al., 2013). Interestingly, while the N-terminal domain of the a1 subunit carries a typical di-acidic motif (EE--D) for COPII-mediated export, in those of the a2 and a3 isoforms there is none. Moreover, in a β-AP3 knock-out mutant (Feraru et al., 2010) VHA-a3 was detected as normal at the tonoplast (Viotti et al., 2013), and its transport was not stopped at the level of intermediate compartments by using the post-Golgi-transport inhibitor concanamycin A (ConcA). Similarly, the second *Arabidopsis* vacuolar proton pump, the H^+^-PPase AVP1, did not accumulate to the Golgi/TGN interface upon ConcA treatment, and it did not localize to the limiting membrane of MVBs (Viotti et al., 2013). In other words, none of the known Golgi- and post-Golgi trafficking routes seemed to be involved in the delivery of the two vacuolar proton pumps.

AVP1 was not only detected to the limiting membrane of rounded vacuoles, but also uniformly present on the membranes of lytic vacuole precursors, the provacuoles. Provacuoles display a much finer (down to 30 nm thickness) tubular network in provascular cells of the root meristem, they are acidic, they carry VHA-a3 too, can fuse with already-developed vacuoles, and are distinct structures respect to autophagosomes (Viotti et al., 2013).

How is AVP1 transported from the ER to the provacuole and where does the latter originate from? A hypothesis is provided in the last section on this review.

## MECHANISMS OF VACUOLE BIOGENESIS

Relatively little is known about the biogenesis of vacuoles in plants. Even the donor membrane from where newly formed vacuoles originate from is unclear. The model that boasts most of the credits in text books suggests that newly formed lytic vacuoles in root-tip cells originate from post-Golgi-derived vesicles (Marty, 1999; De, 2000; Robinson and Rogers, 2000). These vesicles would homotypically fuse to form tubular structures that represent the precursors of vacuoles, the provacuoles. The tubular provacuoles are supposed to fuse with one another, forming a complex network that finally will give raise to the central vacuole (Marty, 1999). This hypothesis is based on an early electron microscopy study that revealed tubular structures at the *trans*-side of the Golgi apparatus which were strongly electrondense after incubation with sodium β-glycerophosphate or cytidine 5^′^-monophosphate (Marty, 1978). These two compounds serve as substrates to detect acid phosphatase and thiolacetic acid esterase activity respectively, thus they were used as biochemical markers to highlight acidic compartments. The tubular-vesicular structure at the *trans*-side of the Golgi was named Golgi-associated endoplasmic reticulum (GERL; Marty, 1978), and later it was renamed as TGN (Griffiths and Simons, 1986). Due to its acidic intraluminal pH, the TGN was proposed to represent the donor membrane for the biogenesis of the lytic vacuole (Marty, 1999).

A few years ago it was shown in *Arabidopsis* that the vacuolar H^+^-ATPase localizes also to the TGN, thus it is not a purely “vacuolar” enzyme. The V-ATPase (VHA) is a holoenzyme composed by two subcomplexes: the membrane-integral complex V_0_, and the cytosolic complex V_1_, both composed by multimeric subunits (Schumacher and Krebs, 2010). The subcellular localization depends on which isoform of the “a” subunit is incorporated in the V_0_ subcomplex. Enzymes incorporating a1 are exclusively located to the TGN, instead enzymes incorporating either a2 or a3 localize to the tonoplast (Dettmer et al., 2006). The presence of VHA-a1 at the TGN contributes to the acidification of tubules at the *trans*-side of the Golgi, and this might explain Marty’s (1978) data. However, conclusive experimental evidence to unequivocally prove that the TGN represents the donor membrane for the biogenesis of the vacuole is lacking up to date.

## THE ER IS THE MAIN MEMBRANE SOURCE FOR VACUOLE BIOGENESIS

Immunogold electron microscopy found AVP1-positive provacuoles directly connected to the ER, and immuno-fluorescent *in situ* visualization of sterols showed that ER-export of newly formed membranes can be COPII-independent in *Arabidopsis* roots (Viotti et al., 2013).These data point to the presence of an unknown mechanism in the endoplasmic reticulum that incorporates VHA-a3 and AVP1 to the provacuolar membrane that arises from the ER (**Figure 1**). It is important to mention that this mechanism could not occur anymore in fully mature cells where the growth of the central vacuole and the turnover of tonoplast resident proteins would involve only Golgi- and post-Golgi trafficking.

The hypothesis that the ER was the membrane source for the biogenesis of the vacuole was already proposed decades ago by Matile and Moor (1968) after ultrastructural analysis via freeze-etching of *Zea mays* seedlings, and one year later Mesquita (1969) published intriguing electron-micrographs showing connections between vacuoles and the rough ER in *Lupinus albus* roots. During the 1980s, other studies reproposed the ER to be the donor compartment for vacuolar biogenesis (Amelunxen and Heinze, 1984; Hilling and Amelunxen, 1985), nevertheless the absence of immunocytochemistry in these old works did not allow an univocal determination of structures’ identity.

**FIGURE 2:**
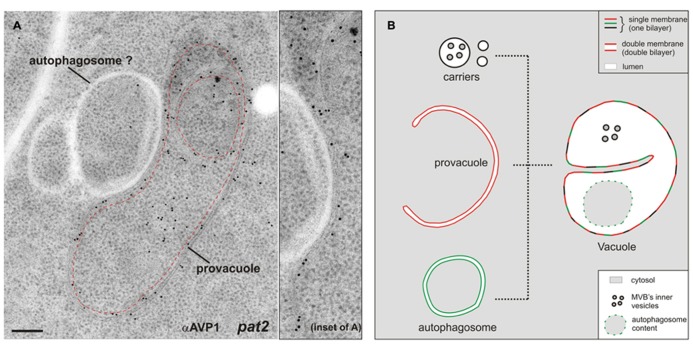
**(A)** Immunogold labeling on ultra-thin sections of high-pressure frozen, freeze-substituted, and HM20-embedded *Arabidopsis* *pat2* root-tip cells shows a provacuole (dashed red line) carrying the V-PPase AVP1. Adjacent to the provacuole, a ring-like structure which is not labeled by the antibody (αAVP1) could be an autophagosome. This picture shows that at least two distinct populations of circular/semicircular double-bilayered structures existin *Arabidopsis* root-tip cells. Scale bar, 200 nm. **(B)** The development of mature vacuoles is probably the result of fusion events between provacuoles, autophagosomes and vacuolar transport carriers.

The *Arabidopsis* gene *VACUOLESS 1* (*VCL1*) is crucial for vacuole development, since embryo and suspensor cells in the *vcl1* knock-out mutant do not develop vacuoles and the mutant is embryo-lethal (Rojo et al., 2001). Interestingly in this study the authors reported the presence of a high number of auotophagosome-like structures in the embryo cells, which could have been, at least partially, provacuoles. From an ultrastructural point of view provacuoles appear indeed similar to autophagosomes. A recent study nicely depicted autophagosomes at the ultrastructural level for the first time in plants (Zhuang et al., 2013). Provacuoles and autophagosomes seem to be distinct entities, since the former were normally found in *atg2*, *atg5*, and *atg7* knock-out mutants, which lack these key players for the formation of the phagophore (Viotti et al., 2013). Since immunocytochemistry of AVP1 did not label several ring-like structures either in wild-type or in *pat2* seedlings (**Figure 2A**), this data point to the presence of distinct populations of circular double-bilayered membranes in the root meristem of *Arabidopsis*. It is likely that provacuoles and autophagosomes might at some point fuse, both contributing to the development of the vacuole (**Figure 2B**).

Several Golgi- and post-Golgi-trafficking mutants, such as *pat2*, *vps45*, and *amsh3*, display alterations in vacuole morphology (Zouhar et al., 2009; Feraru et al., 2010; Isono et al., 2010), and in all these mutants provacuoles were still present albeit often with aberrant profiles (Viotti et al., 2013). The Golgi and the TGN do not seem to be the donor compartments for provacuole formation, but seem to be required for a rapid and efficient development of the vacuolar lumen, and for the delivery of some, although not all, tonoplast resident proteins. The presence of multilayered provacuoles in the *pat2* and *vps45* mutants suggests that an impaired Golgi- and post-Golgi-trafficking could affect the release of provacuoles from the ER, or the fusion between provacuoles and vacuoles, with the result of a proliferation of membranes that at some point start to curl concentrically forming multilayered compartments.

The incorporation of VHA-a3 and AVP1 already in nascent provacuoles at the ER could be explained through the immediate necessity of acidification of the lumen, which is a key feature of vacuolar activities. The molecular players involved in this putative mechanism of sorting and biogenesis are unknown. The functions of VHA-a2, VHA-a3 and AVP1 seem not to be required, since provacuoles and vacuoles are normally present in *vha-a2/vha-a3* and *avp1* mutants (Viotti and Schumacher, unpublished data). Since α-TIP transport in tobacco leaf cells was shown to be BFA-insensitive (Gomez and Chrispeels, 1993) and can be blocked by SEC12 overexpression (Bottanelli et al., 2011), it cannot be excluded that α-TIP follows the same route of VHA-a3 and AVP1, with SEC12 playing an additional role in this process. While we propose that newly formed lytic vacuoles arise from the ER, a subpopulation might originate from the conversion of protein storage vacuoles (PSVs) when seeds start to germinate (Zheng and Staehelin, 2011). Interestingly, direct transport from the ER to PSVs was reported in pumpkin cotyledons and seeds, where precursors-accumulating vesicles (PAC) arise from the endoplasmic reticulum and are delivered to PSVs (Hara-Nishimura et al., 1998). It is tempting to imagine PACs (diameter of 200–400 nm) being the precursors of PSVs, that slowly acquire their final size and identity via Golgi- and post-Golgi-mediated transport, as we have proposed to happen between provacuoles and lytic vacuoles. The idea that membraneous sheets (as provacuoles look like) arise from the ER (that also has similar structures, the cisternae) appears more reasonable in terms of geometry. One of the elements that could contribute to the formation of provacuoles is the different lipid composition of the nascent membrane compared to that of the ER. Theoretically, the clustering in discrete domains of one or more specific kind of lipids could drive the maturation of an organelle from another one. This could also be the case for the maturation of MVBs, which are enriched in phosphatidylinositol-3-phosphate (PI3P), while the Golgi and TGN mainly have PI4P (Vermeer et al., 2006; Vermeer et al., 2009).

More and more evidence is accumulating for direct ER-to-vacuole transport, and those that were supposed to be the “farest” intracellular compartments in plant cells could be, although briefly in time and space, even physically attached.

## Conflict of Interest Statement

The author declares that the research was conducted in the absence of any commercial or financial relationships that could be construed as a potential conflict of interest.
